# A dynamic coordination of flagellum and cytoplasmic cytoskeleton assembly specifies cell morphogenesis in trypanosomes

**DOI:** 10.1242/jcs.166447

**Published:** 2015-04-15

**Authors:** Jack D. Sunter, Vladimir Varga, Samuel Dean, Keith Gull

**Affiliations:** Sir William Dunn School of Pathology, South Parks Road, Oxford OX1 3RE, UK

**Keywords:** Trypanosomes, Morphogenesis, Flagellum attachment zone

## Abstract

Plasma membrane-to-plasma membrane connections are common features of eukaryotic cells, with cytoskeletal frameworks below the respective membranes underpinning these connections. A defining feature of *Trypanosoma brucei* is the lateral attachment of its single flagellum to the cell body, which is mediated by a cytoskeletal structure called the flagellum attachment zone (FAZ). The FAZ is a key morphogenetic structure. Disruption of FAZ assembly can lead to flagellum detachment and dramatic changes in cell shape. To understand this complex structure, the identity of more of its constituent proteins is required. Here, we have used both proteomics and bioinformatics to identify eight new FAZ proteins. Using inducible expression of FAZ proteins tagged with eYFP we demonstrate that the site of FAZ assembly is close to the flagellar pocket at the proximal end of the FAZ. This contrasts with the flagellum, which is assembled at its distal end; hence, these two interconnected cytoskeletal structures have distinct spatially separated assembly sites. This challenging result has many implications for understanding the process of cell morphogenesis and interpreting mutant phenotypes.

## INTRODUCTION

Plasma membrane-to-plasma membrane connections are common features of eukaryotic cells – either between different portions of the same cell or in intercellular junctions. Cytoskeletal frameworks below each respective membrane secure these connections. A major question arises as to how the assembly of such frameworks is coordinated in the respective cellular compartments in order to provide a coherent membrane-to-membrane junction.

*Trypanosoma brucei* is a protozoan parasite that causes human African sleeping sickness. Trypanosomes are single cells with a distinctive form and shape that is inextricably linked to pathogenesis. This shape is defined by an internal sub-pellicular microtubule-based cytoskeleton and a single flagellum that is attached to the cell body for the majority of its length. The lateral attachment of the flagellum is mediated by the flagellum attachment zone (FAZ), which is a large cytoskeletal structure that has a key role in cell morphogenesis; perturbations of the FAZ lead to dramatic changes in cell shape and form that are often lethal. Furthermore the FAZ shows remarkable structural similarity to desmosomes, which are crucial for cell–cell adhesion in multicellular organisms. A greater knowledge of the components and assembly of the FAZ is essential for understanding its role in the morphogenesis of the trypanosome cell.

The *T. brucei* flagellum extends from a basal body that is attached to the kinetoplast (mitochondrial DNA) (Gluenz et al., 2011; Ogbadoyi et al., 2003; Robinson and Gull, 1991). The flagellum emerges near the posterior end of the cell body through the flagellar pocket, an invagination of surface membrane at the base of the flagellum, and is then attached to the cell body for the majority of its length. Definition of the flagellar pocket is important because this area of differentiated surface membrane is a major feature in the pathogenicity of the parasite (Field and Carrington, 2009; Gadelha et al., 2009). Attachment of the flagellum is mediated by specialised structures within the FAZ (Hayes et al., 2014; Vaughan et al., 2008) encompassing three major regions, including the two membranes (flagellum and cell body), the fibres that extend from the axoneme and paraflagellar rod (PFR) to the flagellar membrane, the *macula adherens* (or staples) that maintain the attachment between the flagellar membrane and the cell body plasma membrane, and the FAZ filament and associated microtubule quartet (MTQ) within the cell body and the connections from them to the cell body plasma membrane (Höög et al., 2012; Robinson et al., 1995; Sherwin and Gull, 1989; Vickerman, 1969).

The MTQ is nucleated near the pro basal body, wrapping around the flagellar pocket before joining the FAZ and extending along the length of the cell to the anterior pole (Lacomble et al., 2009). The MTQ is antiparallel to the rest of the cohort of microtubules in the sub-pellicular array (Robinson et al., 1995). The cytoplasmic FAZ filament begins above the flagellar pocket and runs parallel to the MTQ (on the left of the MTQ when viewed from the proximal end) (Lacomble et al., 2009). A row of evenly spaced (∼70 nm apart) junctional complexes termed *macula adherens* is arranged along the line of the FAZ filament maintaining the adhesion between the cell body and flagellum (Höög et al., 2012; Vickerman, 1969). The *macula adherens* consist of a central plate structure located between the flagellar and cell body membrane with fibrous connections that extend to both membranes and continue into the flagellum and cell body (Höög et al., 2012; Vickerman, 1969).

Organisation of the basal body, the flagellum and FAZ are crucial for cell morphogenesis and organelle duplication, and these structures are linked to many of the single copy membranous organelles within the cell (Gheiratmand et al., 2013; Hayes et al., 2014; Kohl et al., 2003; Vaughan et al., 2008). The single mitochondrion is segregated by the basal bodies through the tripartite attachment complex (Ogbadoyi et al., 2003). The bilobe is a cytoskeletal structure located at the proximal end of the FAZ filament (Esson et al., 2012) and is involved in Golgi positioning and biogenesis (He et al., 2005).

The FAZ therefore represents one of the most complex plasma membrane-to-plasma membrane junctional complexes described to date in eukaryotic cells. It also represents a subset of such junctional complexes, given that it involves a connection between two areas of plasma membrane within the same cell, in contrast to cell–cell junctions. The initial molecular characterisation of FAZ components was achieved using monoclonal antibodies raised against complex mixtures of *T. brucei* cytoskeletons, and sera derived from infected cattle (Müller et al., 1992; Vaughan et al., 2008). Use of these reagents in protein expression libraries has established the molecular identity of the components such as FAZ1, a FAZ filament protein (Vaughan et al., 2008). Knockdown of FAZ1 by RNA interference (RNAi) causes errors in flagellum attachment and nucleus mis-segregation, but an overall filament structure was maintained (Vaughan et al., 2008). Ablation of another FAZ filament protein, CC2D, inhibits the assembly of the FAZ resulting in detachment of the flagellum along its entire length and major morphological defects leading to cell death (Zhou et al., 2011). Recently, two new components of the cytoplasmic FAZ structure have been identified – Tb927.7.3330 and Tb927.4.5340 – during a search for bilobe components, demonstrating the close proximity of the bilobe and FAZ (Morriswood et al., 2013).

The first membrane protein component of the FAZ to be identified in *T. brucei* was FLA1 through identity with GP72 from *Trypanosoma cruzi* (Nozaki et al., 1996). When *GP72* was knocked out in *T. cruzi* the cells had a detached flagellum and a shorter cell body but were able to proliferate (Cooper et al., 1993). When FLA1 was ablated in *T. brucei* by RNAi the flagellum became detached, resulting in morphological changes that led to cytokinesis errors and death (LaCount et al., 2002). A FLA1-binding protein (FLA1BP) was identified and has been shown to localise to the flagellar membrane (Sun et al., 2013). Interestingly, FLA1 localises to the cell body membrane, so the interaction of FLA1 and FLA1BP might contribute to the connection between the flagellum and cell body. Upon knockdown of FLA1BP expression there was flagellum detachment and a reduction in cell body size, as anticipated, but the cells were viable (Sun et al., 2013).

Two proteins, FLAM3 and ClpGM6, localise to the filaments connecting the axoneme and PFR to the flagellar membrane; therefore, proteins within all three major regions of the FAZ structure have been identified (Hayes et al., 2014; Rotureau et al., 2014). Upon depletion of FLAM3 the flagellum becomes detached, inhibiting FAZ assembly, which leads to morphological changes and finally cell death (Rotureau et al., 2014). ClpGM6 ablation causes a dramatic morphological change with the cells transformed from a trypomastigote to an epimastigote-like shape (Hayes et al., 2014). Unusually for a FAZ protein, after its depletion by RNAi the cells were viable and the epimastigote-like shape was maintained over many generations in culture (Hayes et al., 2014).

The FAZ is a key morphogenetic structure of trypanosomes and its length correlates with cell body length, indicating that there is coordination between FAZ construction and sub-pellicular microtubule assembly (Robinson et al., 1995; Zhou et al., 2011). During each cell cycle a new flagellum and associated FAZ are duplicated, with the anterior end of the new FAZ defining the point of cytokinesis furrow ingression (Robinson et al., 1995). Hence, the FAZ provides a positional link between the posterior basal body, kinetoplast and flagellar pocket region of the cell, and the anterior site of furrow ingression, ensuring the faithful segregation of the single copy organelles, including the flagellum, between the two daughter cells. Furthermore, as shown by the shape change caused by the loss of ClpGM6 and the change in FAZ protein levels during differentiation in the tsetse fly, the FAZ has a crucial role in determining and maintaining cell morphology during the life cycle (Hayes et al., 2014; Rotureau et al., 2011).

The assembly of the new flagellum and PFR occurs at the distal tip with material transported to the site of construction by the intraflagellar transport (IFT) system (Bastin et al., 1999; Farr and Gull, 2009; Kohl et al., 2003). New flagellum assembly begins with the probasal body maturing and docking to the flagellar pocket membrane, thereby allowing the extension of a new flagellar axoneme into the flagellar pocket. The new basal body and short, extending axoneme then rotate around the old flagellum, after which the new flagellum continues to extend alongside the old flagellum until it reaches the stop point (Lacomble et al., 2010). Once the stop point has been reached, the new flagellum continues to assemble but no longer extends towards the anterior of the cell; instead there is elongation of the posterior of the cell, which coincides with a rapid increase in the inter basal body distance (Davidge et al., 2006). FAZ filament assembly initiates along with new axoneme extension and follows the construction of the flagellum but at a slower rate (Kohl et al., 1999). Importantly, FAZ filament construction begins before that of the PFR, implying that the structures required to maintain flagellum adhesion are assembled before the new flagellum has extended out of the flagellar pocket (Kohl et al., 1999).

Trypanosomes have a highly ordered cellular architecture and, combined with their well-annotated genomes and extensive genetic tools, provide an excellent model for studying the high-order assembly of supra-molecular structures (Berriman et al., 2005; Kelly et al., 2007; LaCount et al., 2000; Poon et al., 2012; Sunter et al., 2012). Here, we combined proteomic and bioinformatic approaches to identify eight new FAZ proteins. Using inducible expression of eYFP-labelled FAZ proteins we determined the relationship between the addition sites of these proteins into the FAZ domains. We propose a model to account for the surprising conclusion that the assembly of the FAZ occurs at the proximal end of the structure in the posterior flagellar pocket region; this indicates that the FAZ is constructed with the opposite polarity to the flagellum, which is extended at the distal tip at the anterior end of the cell. Our studies show that a dynamic coordination of flagellum and cytoplasmic cytoskeleton assembly specifies cell morphogenesis in trypanosomes. We discuss the implications of this finding for the analysis of mutant phenotypes and assembly models of high-order transmembrane molecular complexes.

## RESULTS

### Identification of new FAZ proteins

**Fig. 1.**
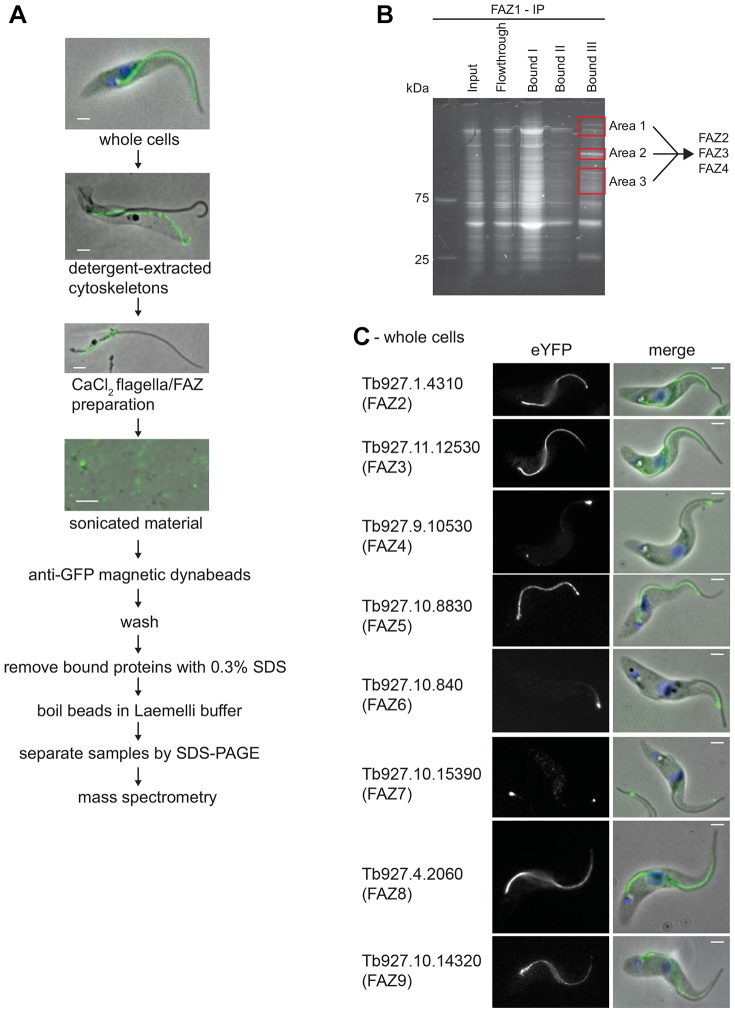
**Workflow for the immunoprecipitation of eYFP–FAZ1 and localisation of new proteins to the FAZ.**(A) eYFP–FAZ1 cell induced for 24 hours with doxycycline, followed by detergent extraction and CaCl_2_ treatment and sonication. Merged images are phase images overlaid with the eYFP–FAZ1 (green) signal. The sonicated material was then treated as outlined in the workflow. (B) Sypro-Ruby-stained SDS-PAGE gel from the FAZ1 immunoprecipitation procedure with bands excised from the gel for analysis highlighted. (C) Cells expressing FAZ–eYFP proteins with eYFP signal and merged images [phase images overlaid with the FAZ–eYFP protein (green) signal]. Scale bars: 2 µm.

A deeper understanding of the FAZ structure and its assembly requires the identification of further components. Here, we identified new FAZ components by tagging FAZ1 with eYFP and using this as a handle for immunoprecipitation, followed by mass spectrometry. A cell line was created with an inducible *eYFP–FAZ1* transgene; on induction eYFP*–*FAZ1 was expressed and localised along the FAZ (Fig. 1). As eYFP*–*FAZ1 localised correctly, this cell line was used for our proteomic approach to identify FAZ components (Fig. 1).

Cells expressing eYFP*–*FAZ1 were extracted with detergent and then isolated flagella with attached FAZs were produced by treatment with CaCl_2_ (Fig. 1). The flagella and FAZ mixture was sonicated and incubated with anti-GFP conjugated beads. After washing, proteins bound to the beads were analysed by mass spectrometry (Fig. 1; supplementary material Table S1). This approach identified proteins that were then endogenously tagged with eYFP (Dean et al., 2015). Three proteins [Tb927.1.4310, Tb927.11.1530 (Tb11.01.4370) and Tb927.9.10530 (Tb09.211.1910) – hereafter termed FAZ2, FAZ3 and FAZ4 (old gene IDs are given in brackets)] localised to the FAZ (Fig. 1C) with distinct but overlapping localisations. FAZ2 was located along the entire length from the flagellar pocket to the anterior end of the cell. The FAZ3 signal started at the proximal end of the FAZ, reducing towards the distal end and absent at the distal tip. FAZ4 was present along the full length but at a low level with an intense signal at the distal end of the FAZ.

Immunoprecipitations were performed on flagella*–*FAZ complexes from eYFP-tagged FAZ2 and FAZ4 cell lines and three further FAZ proteins were identified (supplementary material Table S1): Tb927.10.8830, Tb927.10.840 and Tb927.10.15390 hereafter termed FAZ5, FAZ6 and FAZ7 (Fig. 1C). FAZ5 localised along the length of the FAZ, FAZ6 localised only in the distal portion with a strong signal at the anterior end of the cell body and FAZ7 localised only to the distal end of the FAZ. Importantly, in addition to identifying new FAZ proteins the immunoprecipitation approach resulted in the identification of known FAZ proteins such as CC2D and FLA1BP (supplementary material Table S1).

### Bioinformatic analysis of FAZ proteins

The FAZ proteins were analysed to identify conserved domains or motifs (Table 1). The majority were characterised as being large with predicted coiled coils, repetitive regions and no known domains. However, some did have predicted domains such as FAZ7 with a kinesin domain. A feature shared between four of the FAZ proteins was the presence of a PFAMB506 domain (Pfam ID PB000506). This domain is only found in nine proteins in the *T. brucei* genome: four of these proteins localise to the FAZ and another four (GB4, PFC20, FLAM8, Tb927.10.870), all associate with the cytoskeleton, leaving one uncharacterised protein (Morriswood et al., 2013; Portman et al., 2009; Rindisbacher et al., 1993; Subota et al., 2014; Vaughan et al., 2008). The remaining PFAMB506-domain-containing protein (Tb927.4.2060) was tagged with eYFP at the C-terminus to determine its localisation. Tb927.4.2060 localised along the entire length of the FAZ and hence was termed FAZ8 (Fig. 1C).

**Table 1.**
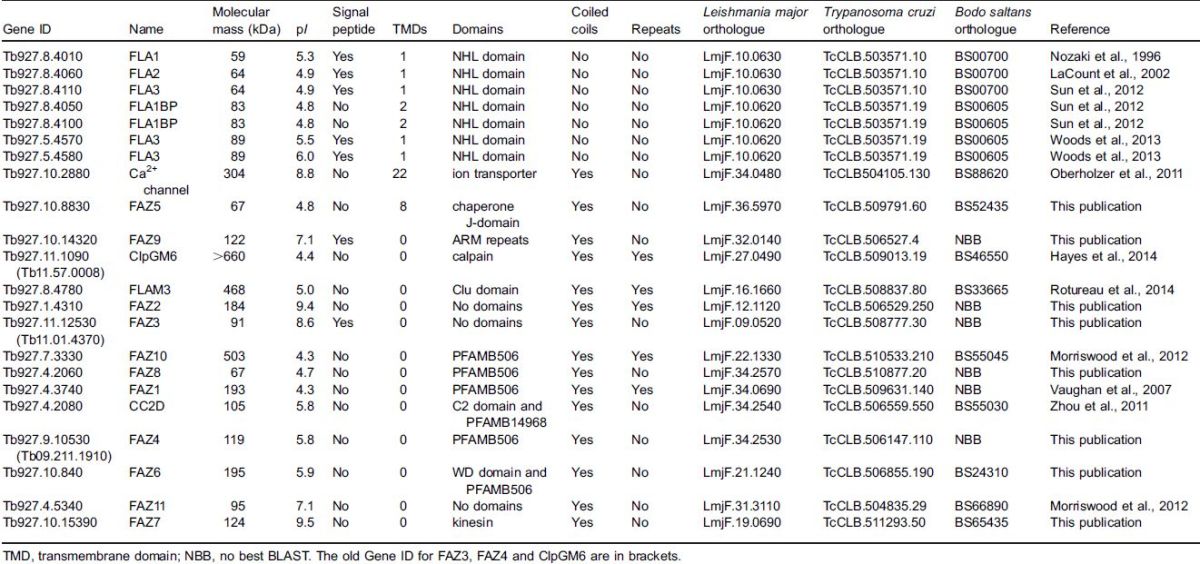
Bioinformatic analysis of the previously identified and newly identified FAZ proteins

Aspects of the FAZ are reminiscent of cell–cell junctions in multi-cellular organisms such as desmosomes. The extracellular core of desmosomes is composed of cadherins, which bind to cadherins found on another desmosomal ‘half unit’ located on an adjacent cell (Garrod and Chidgey, 2008). The cytoplasmic plaque connects the cadherins to the internal intermediate filaments; its main constituents are armadillo family members such as plakoglobin and plakophilin and cytoskeleton adaptor proteins such as desmoplakin (Garrod and Chidgey, 2008). The *T. brucei* genome was interrogated for proteins that contain domains present in desmosomal proteins, such as the cadherin domain and armadillo repeats. No proteins were identified with cadherin domains but three proteins contained armadillo repeats: two of these had previously been characterised (Tb927.6.2640, TbKAP60, a nuclear pore complex protein, and Tb927.1.2670, PF16, a flagellar axoneme protein) so the remaining one, Tb927.10.14320, was tagged at the C-terminus with eYFP (Branche et al., 2006; DeGrasse et al., 2009). Tb927.10.14320 localised to the FAZ, with a stronger signal at the proximal end, which reduced towards the distal end (Fig. 1C). We termed this protein FAZ9.

**Fig. 2.**
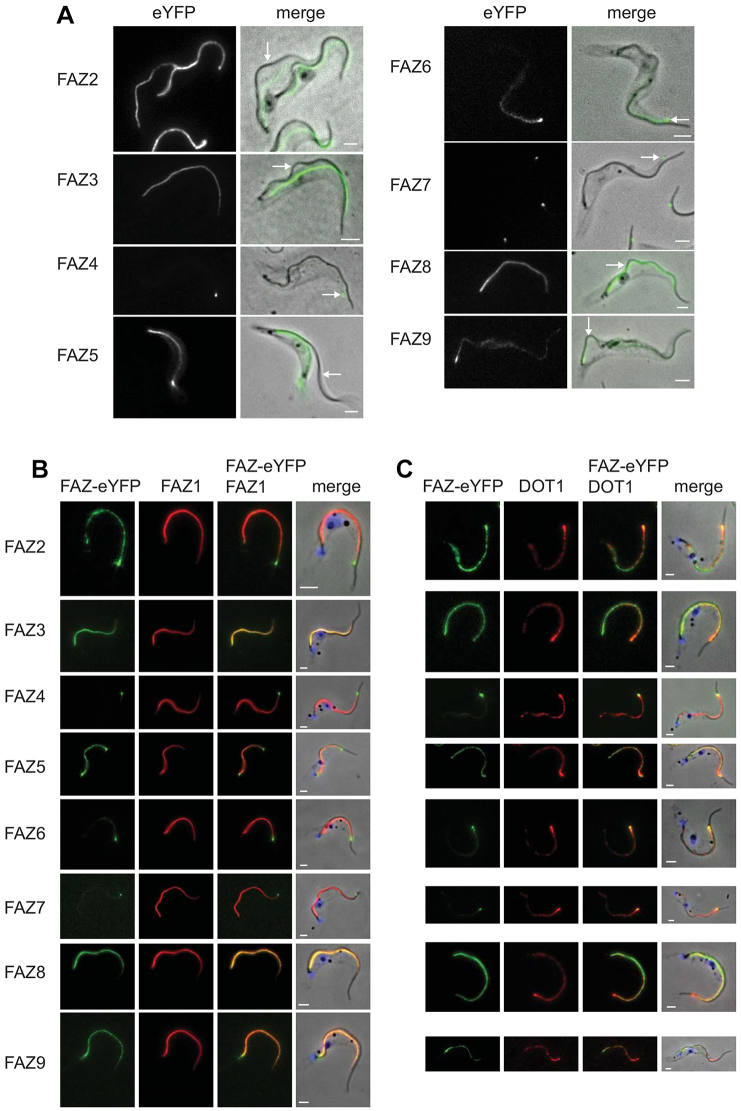
**FAZ proteins are stably associated with the cytoskeleton and colocalise with FAZ markers.**(A) Cytoskeletons from cells expressing FAZ–eYFP proteins. The left images show the eYFP signal and the merged images are phase overlaid with the eYFP (green) signal. Arrows indicate regions where the flagellum is detached and the eYFP signal remains with the cell body. (B,C) Cytoskeletons expressing FAZ–eYFP proteins (green) stained with FAZ1 and DOT1 antibodies (red) respectively. Scale bars: 2 µm.

The majority of the FAZ proteins are conserved in *Leishmania major* and *Trypanosoma cruzi*, and orthologues are present in *Bodo saltans*; however, the FAZ proteins are not found in organisms outside of the kinetoplastids (Table 1). To establish whether the newly identified FAZ proteins were stably associated with the cytoskeleton, the cells were treated with detergent. All eight new FAZ proteins remained associated with the cytoskeleton (Fig. 2A). On cytoskeletons where the flagellum had become detached the FAZ signal remained with the cell body, suggesting that these proteins are components of the cell-body-associated FAZ structure (Fig. 2A). Three of the new FAZ proteins were likely to be components of the membrane zone of the FAZ; FAZ5 has multiple predicted transmembrane domains and FAZ3 and FAZ9 have predicted signal peptides (Table 1). Colocalisation analysis between the new FAZ proteins and two FAZ markers, FAZ1 and DOT1, was performed (Fig. 2B,C). FAZ1 extended along the FAZ with a stronger signal at the proximal end, which reduced towards the distal end, whereas DOT1 had a punctate pattern along the FAZ that was weaker at the proximal end than the distal end. All the new FAZ proteins colocalised with either FAZ1 or DOT1 or both, with the anterior tip signal of FAZ4, FAZ6 and FAZ7 colocalising with DOT1.

### Models for FAZ assembly

After the new flagellum emerges from the flagellar pocket it extends while being attached to the cell body, and assembly of the flagellum and associated FAZ structure occur at relatively similar rates (Kohl et al., 1999). Flagellar axoneme and PFR assembly occur at the distal tip with new components being transported there by the IFT system; however, the site of FAZ assembly is unknown (Bastin et al., 1999; Farr and Gull, 2009; Kohl et al., 2003).

**Fig. 3.**
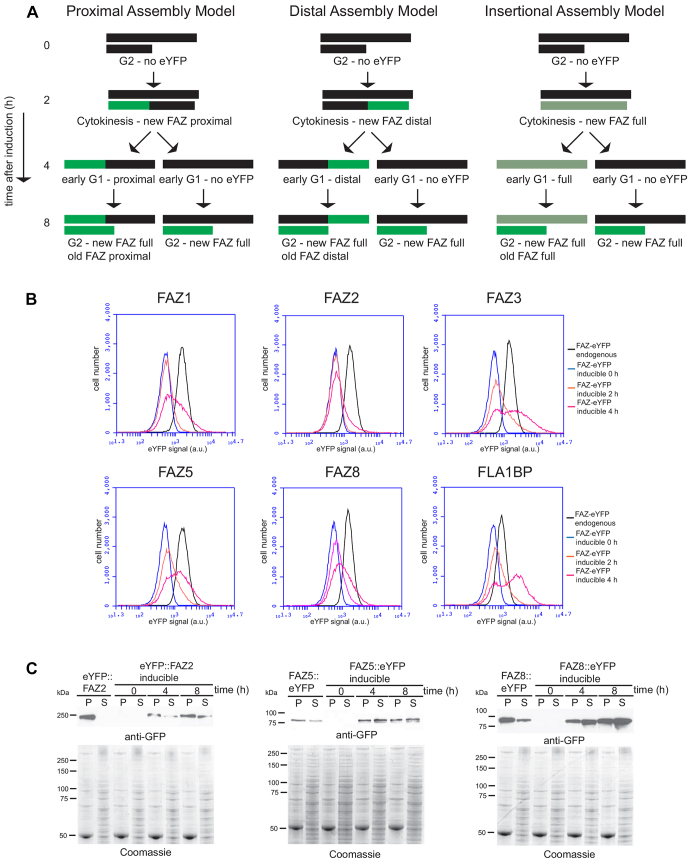
**Models of addition of new FAZ proteins and expression of FAZ–eYFP over the time course.**(A) The cartoons show the pattern of FAZ labelling a G2 cell would produce at each time-point during an induction given the different addition models. A black line represents the FAZ before doxycycline induction; two parallel black lines indicate that the assembly of a new FAZ has begun. A green line represents the addition of FAZ–eYFP proteins to the assembling FAZ after induction. The proximal assembly model shows the position of proximally added FAZ–eYFP proteins during the time course. The distal assembly model shows the position of distally added FAZ–eYFP proteins during the time course. The insertional assembly model shows the position of randomly added FAZ–eYFP proteins during the time course. (B) Expression of FAZ–eYFP proteins measured by flow cytometry for all the endogenously tagged and inducible cell lines (0, 2 and 4 hours post induction). (C) Western blot detecting FAZ–eYFP proteins (FAZ2, FAZ5 and FAZ8) from endogenous tagged and inducible cell lines (0, 4, 8 hours post induction) after detergent fractionation of the cells into pellet (P) and supernatant (S) fractions using anti-GFP antibody. A Coomassie-stained gel acts as a loading control and confirms successful fractionation; 4×10^6^ cell equivalents were loaded per lane.

Three models of FAZ assembly can be envisaged (Fig. 3A; supplementary material Fig. S1): (1) addition of new FAZ proteins to the proximal end of the assembling FAZ, (2) addition of new FAZ proteins to the distal end of the assembling FAZ, or (3) addition of new FAZ proteins inserted randomly along the length of the assembling FAZ. Here we use the term ‘addition’ to mean integration of new FAZ proteins into the assembling FAZ structure, as defined by localisation and resistance to cytoskeleton detergent extraction. The localisation patterns of newly added FAZ proteins after induction of expression were modelled for each of the hypothesised modes of assembly (Fig. 3A; supplementary material Fig. S1). For each model, two assumptions were made: first, that the FAZ was completely assembled in one cell cycle with no addition beyond this; and second, that there was no post-assembly addition of new FAZ proteins to the existing FAZ structures. The models predict that in an assembling FAZ, the FAZ proteins present before induction will be unlabelled and the FAZ assembled after induction will contain the FAZ–eYFP proteins. The location of the FAZ–eYFP proteins therefore indicates the site of FAZ protein addition. The insertional addition model predicts that the addition of FAZ–eYFP proteins occurs randomly along the assembling FAZ. Given that induction is initiated in an asynchronous population of cells with flagella at all stages of extension, this will result in a new FAZ labelled along its entire length with the strength of the signal inversely proportional to the length of the unlabelled FAZ present at the start of the induction. This means that a complete FAZ structure that had only assembled a small section before induction will have a stronger fluorescence signal than a complete FAZ structure that had assembled a longer length of FAZ before induction.

### FAZ protein addition occurs at the proximal end of the FAZ

In order to identify the region of the FAZ in which newly synthesised protein was added, six FAZ genes were cloned into an inducible expression plasmid with an eYFP tag (supplementary material Table S2). The FAZ consists of three regions: fibres radiating from the axoneme to the flagellar membrane, connections across the flagellar and cell body membranes, and the cytoplasmic FAZ filament and associated elements. Ideally proteins found in each zone of the FAZ and that were present along the majority of its length would be analysed; however, the known FAZ components located within the fibres on the flagellum side were too large to be readily cloned. Three proteins from the membrane zone (FLA1BP, FAZ3, FAZ5) and three proteins from the cell body side (FAZ1, FAZ2 and FAZ8) were successfully cloned. Importantly, FLA1BP localises to the flagellar membrane and provides information about the assembly of the flagellum-associated FAZ structure.

**Fig. 4.**
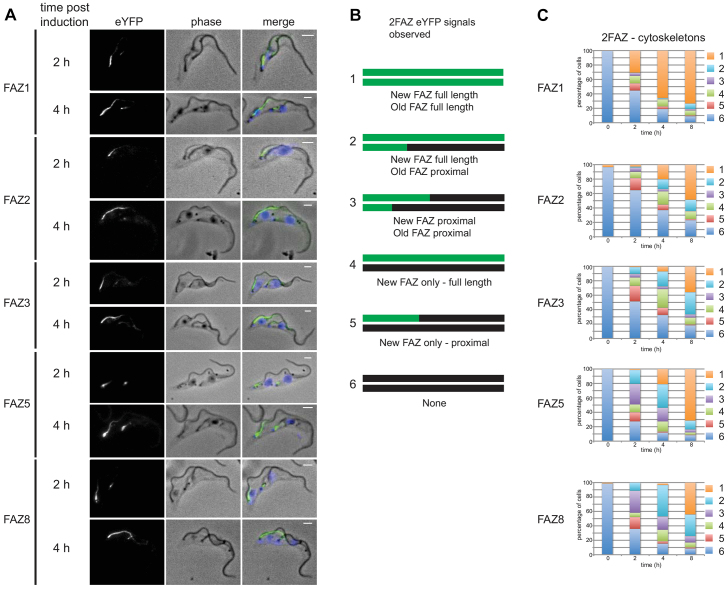
**New FAZ proteins are added at the proximal end of the assembling FAZ.**(A) 2FAZ-cytoskeletons at 2 and 4 hours post induction with phase, the FAZ–eYFP protein signal and merged images [phase overlaid with FAZ–eYFP protein (green) and DAPI (blue)] shown. Scale bars: 2 µm. (B) Cartoons showing the different FAZ–eYFP signals observed in 2FAZ-cytoskeletons. The top line is the new FAZ and the bottom line is the old FAZ with black marking unlabelled FAZs and green marking FAZ–eYFP-labelled FAZs. (C) Graphs showing the number of each cytoskeleton category observed for 2FAZ-cytoskeletons at 0, 2, 4 and 8 hours after induction (*n* = 250 from three independent experiments).

**Fig. 5.**
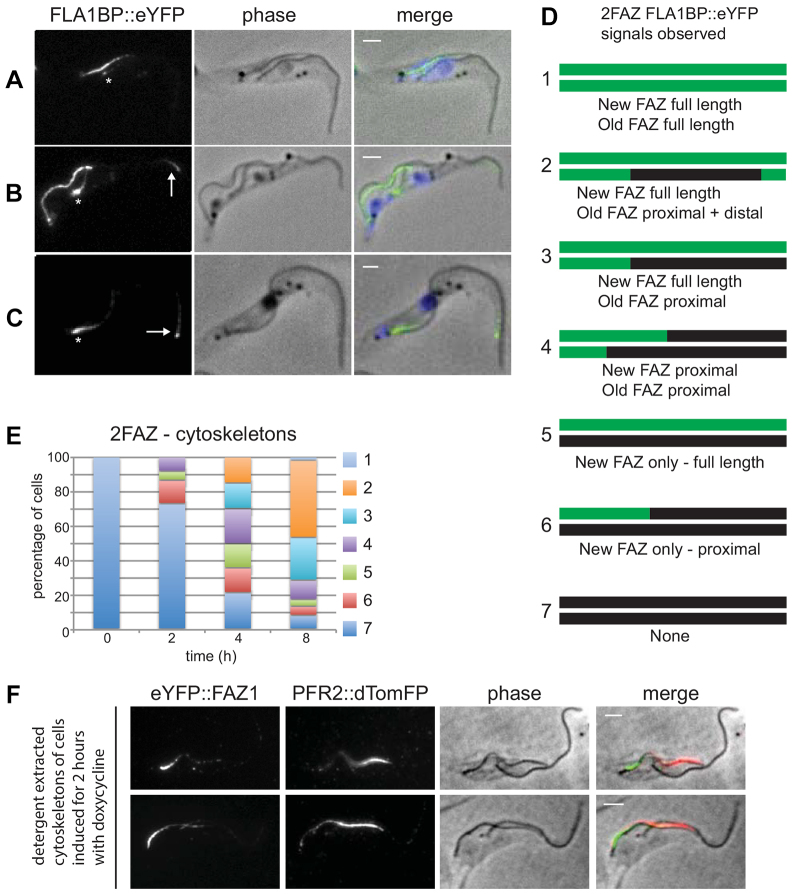
**Newly synthesised FLA1BP protein is predominantly added at the proximal end of the FAZ and the new PFR2 protein is added at the distal end of the flagellum.**(A) 2FAZ-cytoskeleton after 4 hours of doxycycline induction with phase, FLA1BP–eYFP (FLA1BP::eYFP) signal and merged images [phase overlaid with FLA1BP–eYFP (green) and DAPI (blue)] shown. The old FAZ has a proximal signal only (white asterisk). (B) 2FAZ-cytoskeleton after 8 hours of doxycycline induction. The old FAZ has a proximal (white asterisk) and distal signal (white arrow). (C) 1FAZ-cytoskeleton after 8 hours of doxycycline induction. The FAZ has a proximal (white asterisk) and distal signal (white arrow). Scale bars: 2 µm. (D) Cartoons showing the different FLA1BP–eYFP signals observed in 2FAZ-cytoskeletons. The top line is the new FAZ and the bottom line is the old FAZ with black marking unlabelled FAZs and green marking FLA1BP–eYFP-labelled FAZs. (E) Graph showing the number of each cytoskeleton category observed for 2FAZ-cytoskeletons at 0, 2, 4 and 8 hours after induction (*n* = 250 from three independent experiments). (F) Cytoskeletons showing the distinct localisation of FAZ1 and PFR2 in the same cell with phase, the eYFP–FAZ1 signal, the PFR2–dTomFP (PFR2::dTomFP) signal and merged images [phase overlaid with eYFP–FAZ1 (green) and PFR2–dTomFP (red)] shown. Scale bars: 2 µm.

A trypanosome assembles a complete new FAZ within a cell cycle period, with initiation occurring at about the time of G1/S transition (Woodward and Gull, 1990). In an asynchronous *T. brucei* culture, cells are present at every point in the cell cycle. The pattern of addition of the FAZ–eYFP protein into the FAZ after induction in individual cells will reflect the varying patterns of FAZ and new flagellum assembly at particular cell cycle points. We determined the localisation of the FAZ–eYFP proteins in the FAZ at 0, 2, 4 and 8 hours after induction by imaging detergent extracted cytoskeletons (Fig. 4A, Fig. 5A; supplementary material Fig. S2A, Fig. S3A, Fig. S4A).

By way of controls, we ascertained that over the 8-hour time course only a minimal differences in the growth rates and DNA state (kinetoplast nucleus numbers) between the induced and non-induced cell lines were observed (supplementary material Fig. S1D,E). Of the 12,000 cytoskeletons examined over the time course only 17 had a detached flagellum. As a further control, cells expressing eYFP alone were induced: cells expressed increasing amounts of eYFP during the time course but none of the eYFP was retained in the detergent-extracted cytoskeletons (supplementary material Fig. S1F,G).

Before induction, very little background expression was detected – of 3000 cytoskeletons examined only 0.8% had a FAZ–eYFP signal (Fig. 4C, Fig. 5C; supplementary material Fig. S2B, Fig. S3B, Fig. S4B). The induction of the FAZ–eYFP protein expression was analysed by flow cytometry and western blotting (Fig. 3B,C). Flow cytometry enabled FAZ–eYFP protein expression between the inducible and endogenously tagged cell lines to be compared at an individual cell level. The majority of the cells in the 4 h induced populations were either expressing a similar amount of FAZ–eYFP protein or less than that in the equivalent endogenous tagged cell lines, which have a normal phenotype (Fig. 3B). Both the induced and endogenously tagged FAZ–eYFP cell lines were also fractionated with detergent (Fig. 3C and data not shown). There was a soluble pool in some of the endogenously tagged cell lines, which showed a normal phenotype, and overexpression of the FAZ–eYFP protein therefore can also lead to a soluble pool of protein.

The localisation of the FAZ–eYFP proteins after induction was examined in the new FAZ of cytoskeletons from cells with two flagella and hence two FAZs. If new FAZ assembly began after induction, then the entire length of the new FAZ would be labelled with FAZ–eYFP protein; however, if assembly began before induction then the new FAZ would only be labelled selectively where the new FAZ proteins were added. For the cell-body-associated FAZ proteins (FAZ1, FAZ2, FAZ3, FAZ5 and FAZ8) three types of FAZ–eYFP protein patterns were observed in the new FAZs: no eYFP signal, eYFP signal restricted to the proximal end of the FAZ, and eYFP signal along the entire length of the FAZ (Fig. 4A–C; supplementary material Fig. S2A, Fig. S3A, Fig. S4A). As the time course progressed there was an increase in the number of new FAZs labelled along their entire length and a concomitant drop in unlabelled FAZs. The number of proximal labelled new FAZs peaked after 2 hours and was lower at later time points. The localisation patterns observed in 1FAZ-cytoskeletons (i.e. cytoskeletons containing one FAZ) during the time course reflected those observed in the new FAZ of 2FAZ-cytoskeletons (i.e. cytoskeletons containing two FAZs): an increase in cells with eYFP signal along the length of the FAZ, a peak of cells with eYFP signal only in the proximal region of the FAZ that then dropped, and a decrease in cells with no signal (supplementary material Fig S2B, Fig. S3B, Fig. S4B).

We observed no cytoskeletons with distal-only labelling of the new FAZ, and many cytoskeletons had proximal-only labelled new FAZs, implying that the proximal assembly model is most likely. The assembly of the flagellum begins roughly halfway through the cell cycle with FAZ1 assembly beginning slightly before this point; both then continue to the end of the cycle (Kohl et al., 1999; Woodward and Gull, 1990). The model assumed that FAZ assembly was finished within one cell cycle and as the average doubling time of the inducible cell lines was 11.5 hours FAZ assembly is likely to take >5 hours; therefore, the presence of 2FAZ-cytoskeletons with stably integrated FAZ–eYFP proteins in the old FAZ at early post induction timepoints was unexpected and suggested that the assembly of the FAZ was more complex than initially envisaged. We observed 2FAZ-cytoskeletons, where in addition to labelling of the new FAZ, the old FAZ was labelled at the proximal end only. In these cytoskeletons, the assembly of the old FAZ was initiated in the previous cell cycle before FAZ–eYFP induction and the presence of proximal only labelling in these FAZs suggests that the FAZ continues to grow beyond one cell cycle.

Furthermore, at 2 and 4 hours post induction, we also observed 2FAZ-cytoskeletons with the old FAZ labelled along its entire length; this is unlikely to have occurred at these early post induction time points without the post-assembly addition of FAZ-eYFP proteins to the existing old FAZ along its entire length (Fig. 4C). If post-assembly addition to the FAZ had occurred, the number of unlabelled full-length FAZs (i.e. those assembled before induction) would progressively drop after expression of the FAZ–eYFP proteins. To estimate the number of unlabelled full-length FAZs in the population, the number of FAZs present in each localisation category for the 1FAZ-cytoskeletons and the old FAZ in 2FAZ-cytoskeletons at each time point were summed (supplementary material Fig. S2C, Fig. S3C, Fig. S4C). This addition, however, does not account for the continued division of the cells, which would cause a reduction in the proportion of the cells with an unlabelled FAZ. To compensate for cell division, the sum of the FAZs in each category was multiplied by the cell density at that time point to give the inferred number of FAZs: this is an estimate of the total number of full-length FAZs in each category present in the population at that time point. Another factor to take into account is our finding that the old FAZ continues to assemble in the cell cycle after its genesis, as this means that a proportion of the proximally labelled FAZs observed after induction would have been present without any label before induction. Hence, we use the total of the unlabelled and proximally labelled FAZs as the value for the number of unlabelled full-length FAZs assembled before induction remaining at each time point. This measure drops across the time course, suggesting that there was post-assembly addition of FAZ1, FAZ2, FAZ3, FAZ5 and FAZ8 eYFP proteins (supplementary material Figs S2C–S4C).

### FLA1BP addition to the FAZ

After induction, the patterns of FLA1BP–eYFP localisation fell into a similar set of categories to those observed with the other FAZ proteins (Fig. 5A–E; supplementary material Fig. S4). There was addition of FLA1BP to the proximal end of the new and old FAZs in 2FAZ-cytoskeletons, suggesting that FLA1BP follows the proximal addition model. However, we also observed different patterns of FAZ labelling. Some cytoskeletons had FLA1BP labelling at both the proximal and distal ends of the FAZ, with no FLA1BP signal between the two ends (Fig. 5B,C; supplementary material Fig. S4A). This pattern of labelling was present on cytoskeletons with both one FAZ and two FAZs. In the 2FAZ-cytoskeletons the proximal and distal staining was only present on the old FAZ, suggesting that the distal addition of FLA1BP occurred only on full-length FAZs and not during the assembly of a new FAZ (Fig. 5E). Importantly, no cytoskeletons were observed that had FLA1BP signal only at the distal end of the FAZ, bolstering the conclusion that the predominant site of FLA1BP addition was at the proximal end of the FAZ. In contrast to the other FAZ proteins examined, the inferred number of unlabelled and proximally labelled FAZs in the 1FAZ-cytoskeletons and the old FAZ of 2FAZ-cytoskeletons increased slightly with time, indicating that there was no post-assembly addition of FLA1BP into the FAZ (supplementary material Fig. S4C).

### FAZ and flagellum assembly in the same cell

We have previously shown that the PFR and the axoneme are assembled at the distal tip of the growing flagellum (Bastin et al., 1999; Farr and Gull, 2009). To confirm the proximal addition of new FAZ proteins, we constructed a cell line capable of inducible expression of the FAZ1 protein along with the major PFR protein PFR2. The cell line containing both inducible constructs was induced for 2 hours, and cytoskeletons were analysed by microscopy (Fig. 5F). Examination of individual trypanosomes clearly showed a proximal FAZ1 signal and a distal PFR2 signal, demonstrating that new FAZ proteins are added to the assembling structure at the opposite end to new axonemal and PFR proteins.

### FAZ protein depletion by RNAi causes proximal flagellum detachment

**Fig. 6.**
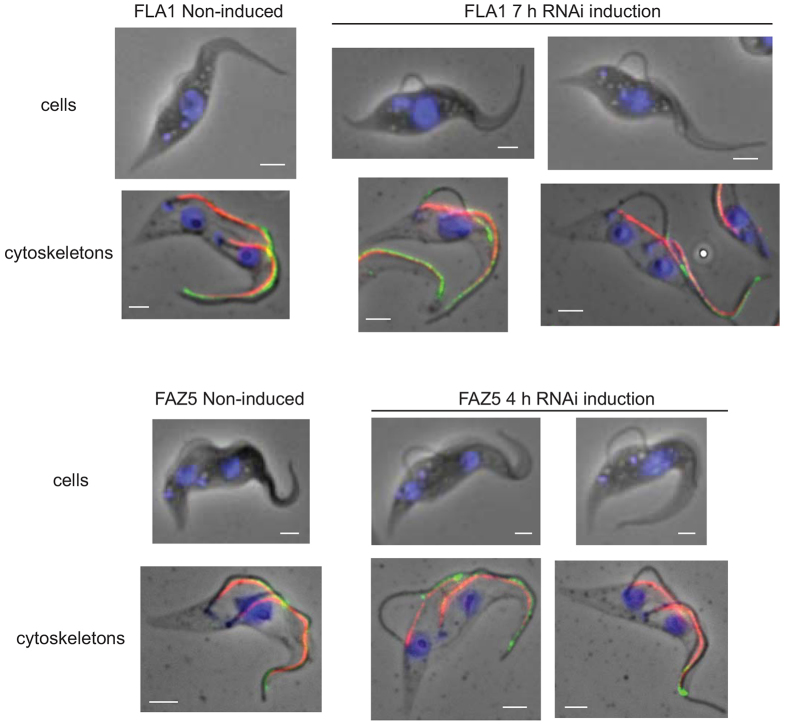
**Induction of FLA1 and FAZ5 RNAi results in loops of detached flagella at the proximal end.**the induction of RNAi-mediated depletion. Cytoskeletons before and after RNAi induction were labelled with anti-FAZ1 (red) and anti-ClpGM6 (green), and DNA was stained with DAPI (blue). The doubling times of the FLA1 and FAZ5 RNAi cell lines were ∼16 h and ∼12 h respectively. Scale bars: 2 µm.

The proximal addition model of FAZ assembly predicts that if the assembly of a new FAZ had begun before the induction of the RNAi against a FAZ protein the distal end of this FAZ structure would be normal; however the proximal end would be deficient in the targeted FAZ protein. In the case of FLA1 RNAi, we would expect to observe cells with a flagellum that was detached towards the flagellar pocket but remained laterally attached to the anterior end of the cell body via a FAZ that had been assembled before RNAi induction. To test this hypothesis, we knocked down either FLA1 or FAZ5 by performing RNAi in cells for a short period of time. After induction, we observed cells with two flagella where the old flagellum was attached normally and the new flagellum was only attached at the distal end with a loop of free, unattached flagellum at the proximal end (Fig. 6).

## DISCUSSION

Using a combination of proteomic and bioinformatic approaches, we have identified eight new FAZ proteins, bringing the number of known FAZ proteins to 18 (Table 1). The majority of proteins identified here are likely to be present in the cell body region of the FAZ, either in the filament itself or in the fibres between the filament and the *macula adherens*. Three of the identified proteins (FAZ3, FAZ5 and FAZ9) might form part of the membrane region of the FAZ. Given the complexity of the FAZ structure, there are likely to be many more components still to be identified. Another aspect of FAZ protein position highlighted in this study is the heterogeneity of their localisation along the length of the FAZ from the flagellar pocket to the anterior pole, suggesting that in addition to the regions defined on a transverse section through the FAZ there are also regional differences along the length of the FAZ.

Here, we used inducible FAZ–eYFP proteins to define the site of FAZ assembly and consistently observed the same pattern with many different proteins. Our examination of the addition of these FAZ proteins to the assembling new FAZ produced a surprising and challenging result. The flagellum and FAZ form part of the same overall structure; however, our data suggest that there are two distinct spatially separated assembly sites for this structure. The axoneme and PFR are assembled at the distal end of the growing flagellum, whereas the associated growing FAZ is assembled at the proximal end. The FAZ is composed of three major zones: the cytoplasmic filament and associated structures; the transmembrane junctional complexes; and fibres within the flagellum. We investigated the addition of a variety of FAZ proteins present in different parts of the structure to determine whether the site of FAZ assembly is consistent for each of the major zones of the FAZ structure. FAZ1, FAZ2, FAZ3 and FAZ8 are all components of the cytoplasmic region of the FAZ, with FAZ5 and FLA1BP forming part of the transmembrane complexes. Moreover, FLA1BP localises to the flagellar membrane side of the FAZ, providing insight into the assembly of the FAZ components within the flagellum.

The proteins from the cytoplasmic and cell body membrane-associated FAZ structures (FAZ1, FAZ2, FAZ3, FAZ5 and FAZ8) showed similar patterns of proximal addition. We did not detect a single example of a distal-only signal after induction of the FAZ–eYFP protein. FLA1BP behaved slightly differently to the other FAZ proteins. The major site of FLA1BP addition was at the proximal end of the new assembling FAZ, as found for the other FAZ proteins, but there were also a large number of cytoskeletons that showed a distal FLA1BP signal in addition to the proximal signal; the distal signal was always accompanied by a proximal signal. The distal signal was only observed in 1FAZ-cytoskeletons and at the old FAZ in 2FAZ-cytoskeletons but never in the growing new FAZ in 2FAZ-cytoskeletons; therefore, it appears that the region at the anterior end of the flagellar-membrane-associated FAZ might be remodelled during the latter period of the cell cycle. We cannot categorically rule out an additional small proportion of random integration along the length of the growing FAZ, but the dominant conclusion is that these regions of the FAZ located in the cytoplasm, cell body membrane and flagellar membrane extend by proximal addition. The proximal addition model was further confirmed by RNAi experiments. The model predicts that if the assembly of a new FAZ had begun before induction of the RNAi-mediated depletion, the distal end of this FAZ structure would be normal, with the proximal end being deficient in the targeted FAZ protein. This situation was observed with the knockdown of both FLA1 and FAZ5, where the distal end of the flagellum was laterally attached to the cell body but the proximal end was unattached.

In addition to the expected labelling of the new FAZ in 2FAZ-cytoskeletons the old FAZ was also labelled with FAZ–eYFP proteins at the proximal end. At the time points early after induction, the old FAZ in a two FAZ-cytoskeleton would have been assembled in a previous cell cycle before induction began, and the presence of labelling suggests that FAZ assembly continues beyond one cell cycle, raising the possibility that the cell body continues to grow and/or remodel after division. This continued assembly of the FAZ matches the situation observed for the trypanosome flagellum, where distal end assembly has been shown to continue beyond a single cell cycle (Farr and Gull, 2009). Another mode of FAZ component addition was also observed with the post-assembly addition of the cytoplasmic and cell body membrane associated FAZ proteins. However, this type of addition was not observed with FLA1BP, which is localised to the flagellar membrane, suggesting that post-assembly addition is restricted to the cell body side. The proximal addition of new FAZ proteins was clearly demonstrated at 2 and 4 hours post induction and at these times the overexpression of FAZ1, FAZ2, FAZ5 and FAZ8 was minimal. FAZ3 and FLA1BP were overexpressed to higher levels; however, the proximal addition of FAZ–eYFP protein was consistently observed in all cell lines. Whilst it is possible that the expression levels and tagging could influence other more complex labelling patterns such as post-assembly addition the uniformity of the results for six proteins is comforting.

**Fig. 7.**
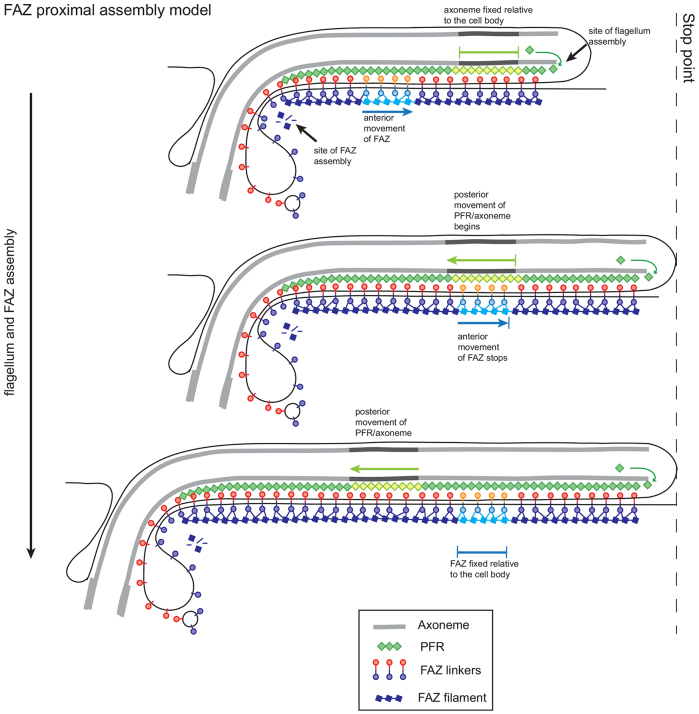
**Model of the assembly of the new FAZ and flagellum during the cell cycle.**, with the relative movement of the flagellum and FAZ indicated.

Using these data we propose a model whereby the FAZ is assembled from its proximal end in a conveyor belt fashion (Fig. 7). Components of the FAZ are transported to the flagellar pocket region, where assembly of the FAZ occurs, and as the FAZ is assembled the older components are moved towards the anterior of the cell, tracking the growth of the new flagellum. The membrane components of the FAZ are trafficked via the flagellar pocket and the flagellar side and cell body side components of the *macula adherens* might interact first in the neck of the flagellar pocket, forming a strong connection that is then built into the FAZ structure. It is possible that some multi-component modules of the FAZ structure might be pre-assembled in the cytoplasm and transported to the site of FAZ construction, mirroring desmosomal assembly (Demlehner et al., 1995).

In multi-cellular organisms, desmosomes create strong cell–cell adhesion by connecting intermediate filaments in two adjacent cells across the plasma membranes (Nekrasova and Green, 2013). Recent advances in desmosomal assembly have relied on time-lapse imaging of fluorescently tagged components, such as the cadherins [desmocadherin-2 (Dsc-2) and desmoglein-2 (Dsg2)] (Nekrasova et al., 2011). Desmosome assembly is a complex multi-step process, which requires interactions between cadherins, armadillo proteins and desmoplakin for correct assembly and adhesive function. Moreover, assembly requires the coordination of components in the membrane and those in the cytoplasmic plaque (Nekrasova and Green, 2013). An initial step in assembly of the desmosome is the transport of Dsc2 and subsequently Dsg2 cadherin-loaded vesicles, being transported to the site of cell–cell contact assembly along microtubules using kinesin motors (Nekrasova et al., 2011). Around the same time as the delivery of Dsg2 vesicles occurs, the assembly of the cytoplasmic plaque begins. First, desmoplakin begins to accumulate at the site of cell-cell contact and then particles containing desmosome components such as desmoplakin and Plakophilin-2 (Pkp2) appear in the cortical region associated with the intermediate filament cytoskeleton (Godsel et al., 2010). Next these components move to the nascent desmosome to strengthen the plaque (Godsel et al., 2005). The final stage of assembly might require Sec3-containing exocyst complexes, which deliver additional cadherins and might be involved in reorganising the cytoskeleton in combination with Pkp2 and the RhoA signalling system (Andersen and Yeaman, 2010; Godsel et al., 2010). The FAZ and desmosomes have analogous structures and functions, but the identified individual components are not conserved. FAZ7 and FAZ9 contain domains (kinesin and ARM repeats) that are important in desmosomal assembly; however, there is no homology to any desmosome protein beyond those conserved domains. Although desmosomal assembly might provide instructive parallels for FAZ assembly, it is important to note that desmosomal components are subject to turnover post-assembly, whereas FAZ proteins appear to be stable once assembled (LaCount et al., 2002; Vaughan et al., 2008; Windoffer et al., 2002).

The new distal tip of the growing FAZ invades, alongside the old FAZ, between the old MTQ and the sub-pellicular microtubules, influencing or responding to the breaking of the connections between them (Wheeler et al., 2013). The polarity of the MTQ is opposite to that of the sub-pellicular microtubules and the asymmetry between the two adjacent microtubules of the respective complexes might help to direct the invasion of the FAZ (Robinson et al., 1995). During the extension of the new FAZ, connections to the new growing flagellum are established that adhere this flagellum to the cell body. The path of new FAZ invasion is determined by the position of the old FAZ; the growth of the new flagellum is therefore guided by the old FAZ as well as by the flagella connector, which, in the procyclic form, connects the tip of the new flagellum to the old flagellum (Moreira-Leite et al., 2001).

The stop point is an important concept in the relationship between flagellum extension and kinetoplast segregation or cell morphogenesis (Davidge et al., 2006; Robinson et al., 1995). In our model, during the assembly of the flagellum and FAZ before the stop point, any given point in the axoneme stays at a fixed position relative to the cell body as assembly occurs at the distal tip of the flagellum, while the proximally assembled FAZ moves towards the anterior pole of the cell body (Fig. 7). After the stop point is reached, the FAZ is now anchored in position and the posterior of the cell elongates, with the previously fixed point in the axoneme now moving towards the posterior of the cell. Taken together, this means that the movement of the flagellum relative to the FAZ does not change during assembly. Furthermore, the fact that there is no change in FAZ localisation patterns at any point of the cell cycle implies that the stop point does not influence FAZ assembly.

The connections between the flagellar skeleton and FAZ are required to maintain the attachment of the flagellum to the cell body over the lifetime of a trypanosome (Ginger et al., 2013); however, the flagellar skeleton is assembled from the distal end, and the FAZ is assembled from the proximal end, raising the issue of when the crucial connections between these two structures are made. Electron microscopy does not reveal any observable changes in the connections between the axoneme and the FAZ in a growing flagellum (Sherwin and Gull, 1989; Vickerman, 1969). It is possible that the fibres connecting the flagellar skeleton and FAZ in an extending flagellum are easily made and broken, as the two structures slide past each other and only once assembly is complete are the connections fully strengthened.

Our tagging and proteomics approach has successfully enabled the identification of new FAZ proteins. Inducible expression of these has led to the discovery that newly synthesised FAZ proteins are added to the proximal end of the assembling FAZ, in contrast to distal polarity seen for the growing flagellum. This new and unexpected finding emphasises the extraordinary complexity of not just the structural components of the FAZ subcomplexes but also their dynamics and coordination of assembly. We suggest a model that involves movement of subcomplexes in the plane of the membranes. The model makes a number of predictions for how subcomplexes might be assembled and moreover has implications for the process of normal cell morphogenesis and interpretation of mutant phenotypes that result in morphogenetic abnormalities.

## MATERIALS AND METHODS

All reagents were purchased from Sigma-Aldrich (Gillingham, UK) unless stated.

### Cells

SMOxP927 and 29-13 FLA1 RNAi cells were grown at 28°C in SDM-79 (Life Technologies, Paisley, UK) supplemented with 10% fetal calf serum (FCS) (Brun and Schönenberger, 1979; LaCount et al., 2002; Poon et al., 2012).

### Tagging, cloning and transfections

eYFP tagging of candidate genes was achieved using a long primer PCR approach (Dean et al., 2015). A fluorescent tag and resistance cassette were amplified from a plasmid template using primers with long 5′ overhangs that target the amplicon to the desired locus.

To create inducible eYFP-tagged FAZ component cell lines, the open reading frames (ORFs) for the FAZ genes were amplified by PCR, and cloned into the pDEX777 (supplementary material Table S2) (Poon et al., 2012). The PFR2::dTomFP inducible plasmid was created by amplifying the coding sequence of PFR2 by PCR and cloning the resulting fragment into the pDEX877 plasmid that carried dTomFP (Poon et al., 2012). Amplifying nucleotides 621–1613 of the FAZ5 ORF and cloning this fragment into pDEX777 twice, in opposing orientation, created the FAZ5 hairpin RNAi plasmid.

The plasmids were linearised by digestion with NotI and cells transfected following a standard protocol with either plasmids or PCR constructs (Dean et al., 2015; Poon et al., 2012). Transgenic cells were selected in presence of either 5 µg/ml phleomycin or 10 µg/ml blasticidin or 25 µg/ml hygromycin. FAZ–eYFP protein expression, PFR2::dTomFP expression and RNAi induction was induced by the addition of doxycycline (1 µg/ml).

### Proteomics

5×10^9^ procyclic cells were harvested by centrifugation (800 ***g*** for 10 minutes), washed in PBS with 5 µM E-64-d and centrifuged as before. The pellet was resuspended in PEME (100 mM PIPES pH 6.9, 1 mM MgSO_4_, 2 mM EGTA, 0.1 mM EDTA) with 1% Igepal CA-630 and protease inhibitors (50 µM leupeptin, 7.5 µM pepstatin A, 500 µM PMSF and 5 µM E-64-d). The mixture was incubated at room temperature for 5 minutes and then centrifuged at 1800 ***g*** for 10 minutes. The pellet was resuspended in ice-cold 20 mM PIPES pH 6.9, 65 mM CaCl_2_ with protease inhibitors and incubated on ice for 30 minutes. The mixture was centrifuged at 5100 ***g*** and 6°C for 17 minutes. The pellet was resuspended in DNase buffer (40 mM PIPES pH 6.9, 5 mM MgSO_4_, 1 mM CaCl_2_ and 2× protease inhibitors) and to this was added an equal volume of DNase I (400 µg/ml) and RNase A (100 µg/ml). The mixture was incubated at room temperature for 5 minutes followed by a centrifugation at 12,200 ***g*** and 6°C for 20 minutes. The supernatant was removed and the pellet washed using PBS with protease inhibitors. The pellet was resuspended in PBS with protease inhibitors and sonicated ten times with 10-second pulses followed by 10 seconds cooling. Anti-GFP-conjugated Dynabeads (Life Technologies, Paisley, UK) were added to the sonicated material and incubated at room temperature for 2 hours. The beads were washed with buffer. The beads were incubated with 0.3% SDS at room temperature for 20 minutes to produce Bound I, followed by 0.3% SDS at room temperature for 5 minutes to produce Bound II, and were then boiled in Laemmli buffer for 5 minutes to produce Bound III. The samples were separated using SDS-PAGE. The analysis of the bound material differed between each immunoprecipitation. For FAZ1, three regions containing distinct bands within the Bound III fraction were analysed with FAZ2, and FAZ3 and FAZ4 were identified as highly abundant hypothetical unknown proteins (supplementary material Table S1). For the FAZ2 immunoprecipitation, the Bound I fraction and flow-through were compared by using normalised label-free quantitative mass spectrometry (Griffin et al., 2010; Trudgian et al., 2011). The difference between the spectral counts of these two fractions was calculated and FAZ5 was identified as a hypothetical protein with a large difference in spectral counts (supplementary material Table S1). For the FAZ4 immunoprecipitation, only the Bound III fraction was analysed. FAZ6 was identified as the most abundant unknown protein. FAZ7 was identified due to its relative abundance and the presence of a kinesin domain (supplementary material Table S1). For western blotting, proteins were resolved by SDS-PAGE, transferred onto nitrocellulose and probed with anti-GFP (Life Technologies, Paisley, UK) and L13D6 antibodies (Kohl et al., 1999).

### Flow cytometry

Live cells were analysed using the BD Accuri C6 flow cytometer with the 533/30 nm filter. 50,000 events were collected for each sample.

### Bioinformatics

Reciprocal best BLAST was used to find orthologous sequences (Altschul et al., 1990). *T. brucei* FAZ protein sequences were used to interrogate the non-redundant protein sequence database on NCBI by BLASTP, which identified sequences in other species. The top hit from each species was then used to interrogate the *T. brucei* genome by BLASTP and if this returned the same *T. brucei* FAZ protein as used in the initial search the two proteins were considered orthologous.

FAZ sequences were analysed by online prediction programs. RADAR (http://www.ebi.ac.uk/Tools/pfa/radar/) to detect repeats in the protein sequences. COILS (http://embnet.vital-it.ch/software/COILS_form.html) to detect the presence of coiled coils (Lupas et al., 1991). Signal peptides and transmembrane domains were predicted using Phobius (http://phobius.binf.ku.dk/). The sequences were used to search the Interpro database (www.ebi.ac.uk/interpro/) and PFAM database (http://pfam.xfam.org). PB000506 domain correct as of February 2013.

### Microscopy

Cells were washed and resuspended in PBS, and settled onto slides. Cells were then either fixed in 4% paraformaldehyde in PBS for 5 minutes, or to extract cytoskeletons, incubated in PEME with 1% Igepal CA-630 for 1 minute. Subsequently, the cytoskeletons were fixed in 4% paraformaldehyde in PEME for 5 minutes. The paraformaldehyde was quenched with 1% glycine in PBS. FAZ1, DOT1 and ClpGM6 were detected using L3B2, DOT1 and anti-ClpGM6 respectively (Hayes et al., 2014; Kohl et al., 1999; Woods et al., 1989). Both the cells and cytoskeletons were incubated with DAPI in PBS for 5 minutes, then washed and mounted with DABCO. A single plane of focus for each cell or cytoskeleton was acquired at room temperature using a Leica DM5500B microscope controlled by the Micromanager software with a 100× objective and an Andor Neo 5.5 sCMOS camera.
